# Muscle Atrophy in Response to Cytotoxic Chemotherapy Is Dependent on Intact Glucocorticoid Signaling in Skeletal Muscle

**DOI:** 10.1371/journal.pone.0106489

**Published:** 2014-09-25

**Authors:** Theodore P. Braun, Marek Szumowski, Peter R. Levasseur, Aaron J. Grossberg, XinXia Zhu, Anupriya Agarwal, Daniel L. Marks

**Affiliations:** 1 Papé Family Pediatric Research Institute, Oregon Health & Science University, Portland, Oregon, United States of America; 2 MD/PhD Program, Oregon Health & Science University, Portland, Oregon, United States of America; 3 Knight Cancer Institute, Oregon Health & Science University, Portland, Oregon, United States of America; 4 Division of Hematology & Medical Oncology, Oregon Health & Science University, Portland, Oregon, United States of America; 5 Department of Internal Medicine, Providence St. Vincent Medical Center, Portland, Oregon, United States of America; University of Rome La Sapienza, Italy

## Abstract

Cancer cachexia is a syndrome of weight loss that results from the selective depletion of skeletal muscle mass and contributes significantly to cancer morbidity and mortality. The driver of skeletal muscle atrophy in cancer cachexia is systemic inflammation arising from both the cancer and cancer treatment. While the importance of tumor derived inflammation is well described, the mechanism by which cytotoxic chemotherapy contributes to cancer cachexia is relatively unexplored. We found that the administration of chemotherapy to mice produces a rapid inflammatory response. This drives activation of the hypothalamic-pituitary-adrenal axis, which increases the circulating level of corticosterone, the predominant endogenous glucocorticoid in rodents. Additionally, chemotherapy administration results in a significant loss of skeletal muscle mass 18 hours after administration with a concurrent induction of genes involved with the ubiquitin proteasome and autophagy lysosome systems. However, in mice lacking glucocorticoid receptor expression in skeletal muscle, chemotherapy-induced muscle atrophy is completely blocked. This demonstrates that cytotoxic chemotherapy elicits significant muscle atrophy driven by the production of endogenous glucocorticoids. Further, it argues that pharmacotherapy targeting the glucocorticoid receptor, given in concert with chemotherapy, is a viable therapeutic strategy in the treatment of cancer cachexia.

## Introduction

Cachexia is a progressive syndrome of weight loss that complicates numerous chronic diseases. Marked by a significant loss of skeletal muscle mass, the presence of cachexia is an independent predictor of mortality in multiple disease states [1], [2]. It is estimated that cachexia is directly responsible for 20% of cancer deaths [3]. Further, declining performance status often precludes more aggressive and potentially curative anti-neoplastic treatment. Numerous preclinical studies have investigated the mechanism by which cancer produces muscle atrophy [4]–[6]. However, relatively few studies have explored the contribution of cancer treatment to the development of cachexia [7].

Systemic inflammation is a common uniting feature of all conditions in which cachexia is present [8]. Inflammatory cytokines are present at increased levels in cancer patients and are believed to convey the catabolic signal via at least two distinct mechanisms [9], [10]. Cytokines act directly on skeletal myocytes to induce muscle atrophy via the engagement of surface receptors and the activation of NFκB [6], [11]. Additionally, inflammatory cytokines activate the hypothalamic-pituitary-adrenal (HPA) axis, resulting in the release of endogenous glucocorticoids [12]. Recently it has become clear that a major component of muscle atrophy in response to systemic inflammation occurs as the result of HPA axis activation by cytokines resulting in the release of glucocorticoids from the adrenal gland [4], [13]. Engagement of the glucocorticoid receptor within the myocyte induces a transcriptional program that leads to the proteasomal degradation of myofibrillar protein. Irrespective of the extracellular origin of the catabolic signal, muscle atrophy occurs via a concerted series of intracellular events culminating in the activation of the ubiquitin-proteasome and autophagy-lysosome systems [14], [15].

Cytotoxic chemotherapy has numerous deleterious consequences to normal tissues, including myelosupression, gastrointestinal toxicity and pulmonary toxicity. Cardiac toxicity is also a well-known side effect of anthracycline chemotherapy and has been the subject of numerous investigations. Anthracyclines produce their biologic activity principally via interaction with topoisomerase II and subsequent double stranded DNA breaks, defects in mitochondrial biogenesis and the production of reactive oxygen [16]. The mechanisms by which cytotoxic chemotherapy affects skeletal muscle, however is less well described. Cisplatin chemotherapy produces significant muscle atrophy in mice, presumably via the activation of NFκB [7]. In addition, chemotherapy administration results in significant systemic inflammation, marked by increased levels of numerous circulating inflammatory cytokines [17]. It is unclear whether this systemic inflammation increases circulating glucocorticoids as both increases and decreases are reported after chemotherapy administration [18], [19]. Therefore we investigated the role of glucocorticoids as a mediator of chemotherapy-induced muscle atrophy by administering CAF (cyclophosphamide, doxorubicin, 5-fluorouracil) chemotherapy to muscle-specific glucocorticoid receptor knockout mice (mGRKO). Our findings demonstrate that a significant component of chemotherapy-induced muscle atrophy depends on glucocorticoid signaling in skeletal muscle.

## Methods

### Ethics Statement

This study was carried out in strict accordance with the recommendations in the Guide for the Care and Use of Laboratory Animals of the National Institutes of Health. The protocol was approved by the Institutional Animal Care and Use Committee of Oregon Health and Science University (Protocol Number: ISO 1731). In all cases, every effort was made to minimize the suffering of experimental animals.

### Animals

**Figure 1:**
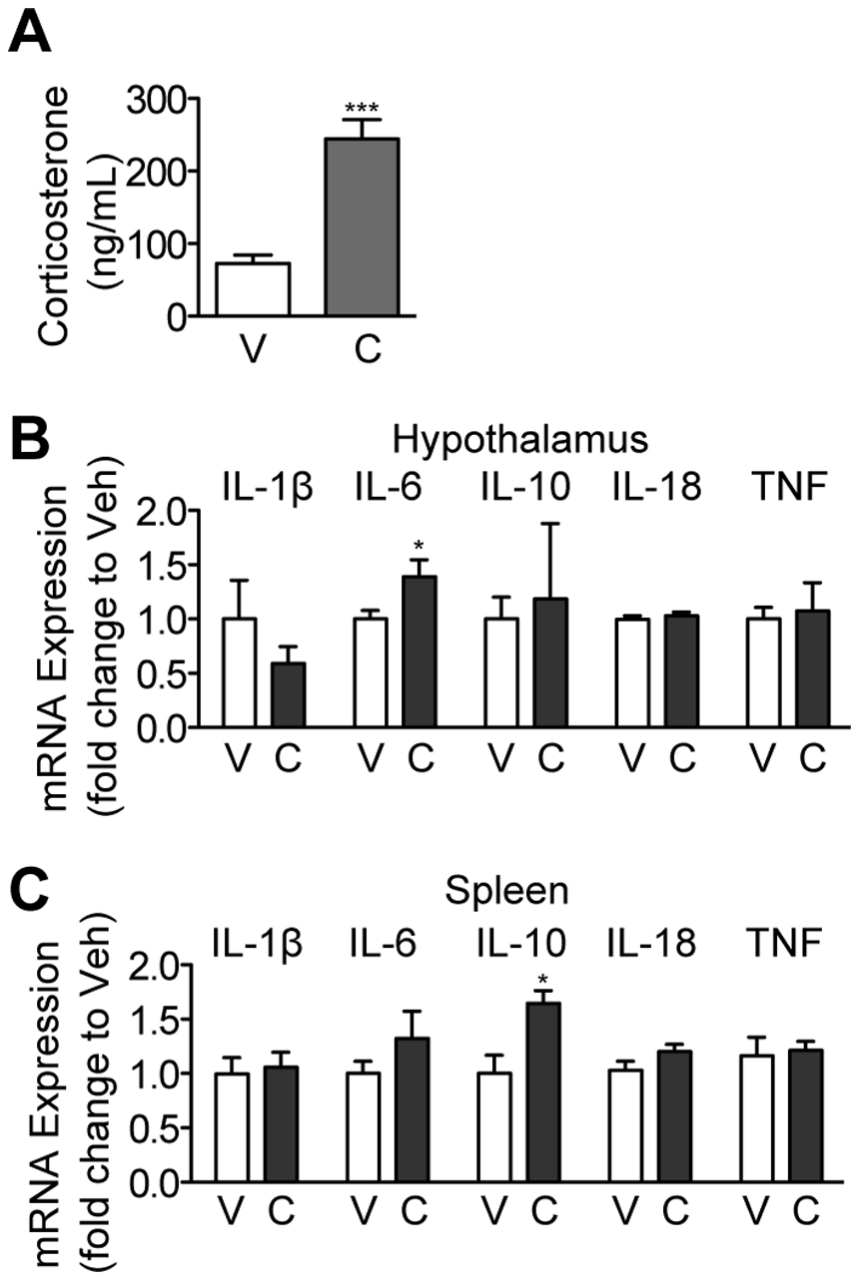
Chemotherapy Administration Increases Circulating Corticosterone and Induces Systemic Inflammation. Wild type mice (8–10/group) were treated with CAF chemotherapy and sacrificed 4 hours later. (A) Plasma corticosterone concentration. (B) Hypothalamic and (C) splenic inflammatory cytokine gene expression was measured by qPCR. * = P<0.05, and *** = P<0.001 vs. sham-treated mice as measured by Student’s t-test. V = Vehicle, C = Chemotherapy.

**Figure 2:**
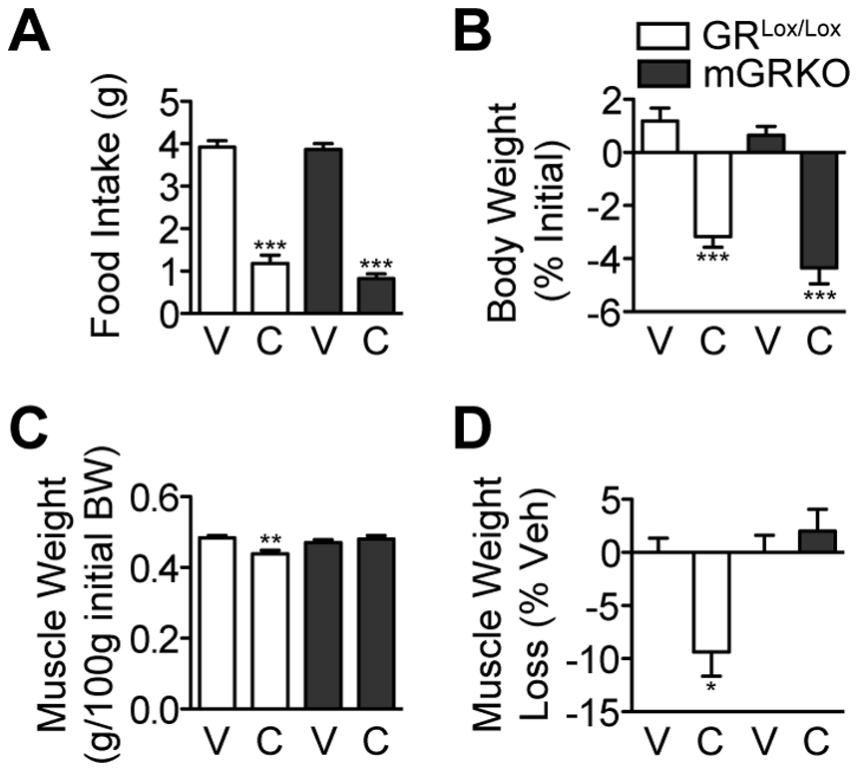
Chemotherapy Administration Produces Muscle Mass Loss in a Glucocorticoid-dependent Manner. GR^Lox/Lox^ and mGRKO mice (n = 6–13/group) were treated with CAF chemotherapy and sacrificed 18 hours later. (A) Food intake and (B) body weight were measured. (C, D) Gastrocnemius muscle was weighed, normalized to pre-treatment body weight and presented as weight loss relative to sham-treated control mice of the same genotype. * = P<0.05, ** = P<0.01, and *** = P<0.001 vs. sham-treated mice of the same genotype as measured by two-way ANOVA with Bonferroni post-test. V = Vehicle, C = Chemotherapy.

**Figure 3:**
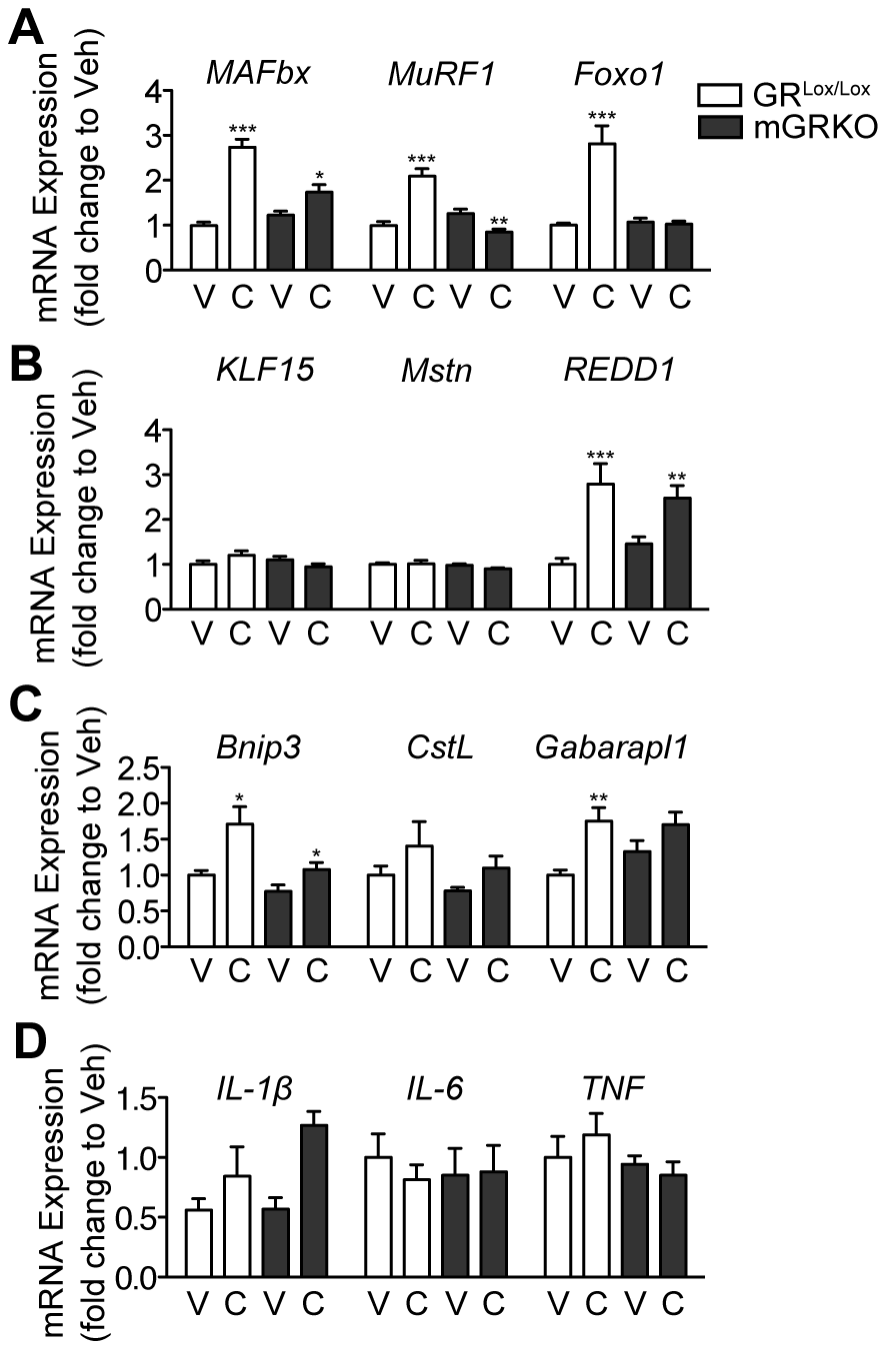
The Induction of Catabolic Gene Expression in Skeletal Muscle Requires Glucocorticoids. GR^Lox/Lox^ and mGRKO mice (n = 6–13/group) were treated with CAF chemotherapy and sacrificed 18 hours later. (A, B, C, D) Gastrocnemius muscle gene expression was measured by quantitative real time PCR with GAPDH as an endogenous control. * = P<0.05, ** = P<0.01, and *** = P<0.001 vs. sham-treated mice of the same genotype as measured by two-way ANOVA with Bonferroni post-test. V = Vehicle, C = Chemotherapy.

**Figure 4:**
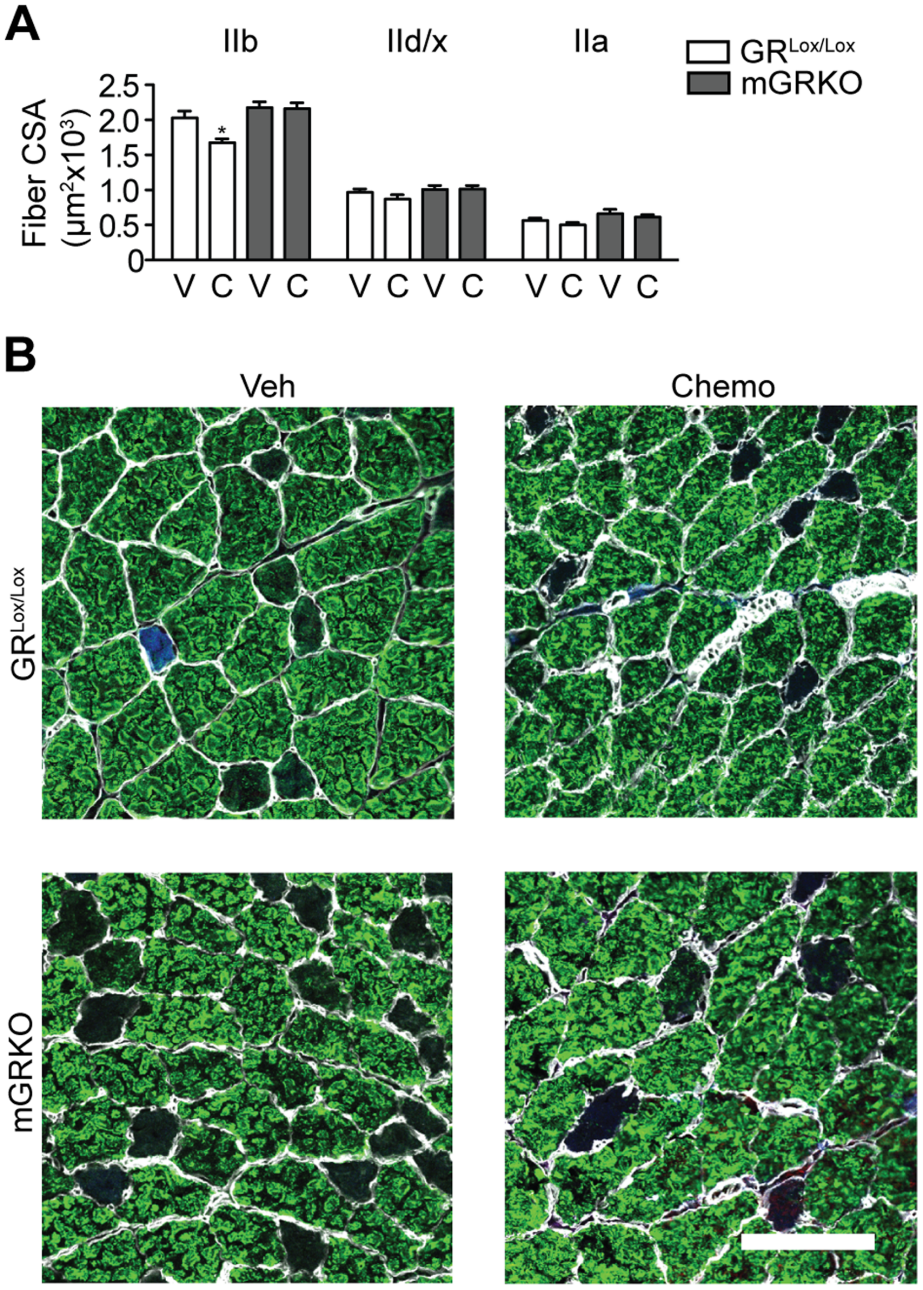
Chemotherapy Produces the Specific Atrophy of Type IIb Muscle Fibers That is Dependent on Glucocorticoids. GR^Lox/Lox^ and mGRKO mice (n = 6–13/group) were treated with CAF chemotherapy and sacrificed 18 hours later. (A) Cryosections of tibialis anterior muscle were immunostained with antibodies to distinct myosin isoforms and the fiber cross sectional area of IIb (green), IIa (blue) and IId/x (black) fibers was measured. (B) Representative images of tibialis anterior muscles that were cut in cross section and immunostained with antibodies against laminin (white), myosin IIb (green) myosin IIa (blue). Scale bar = 100 µM.

**Figure 5:**
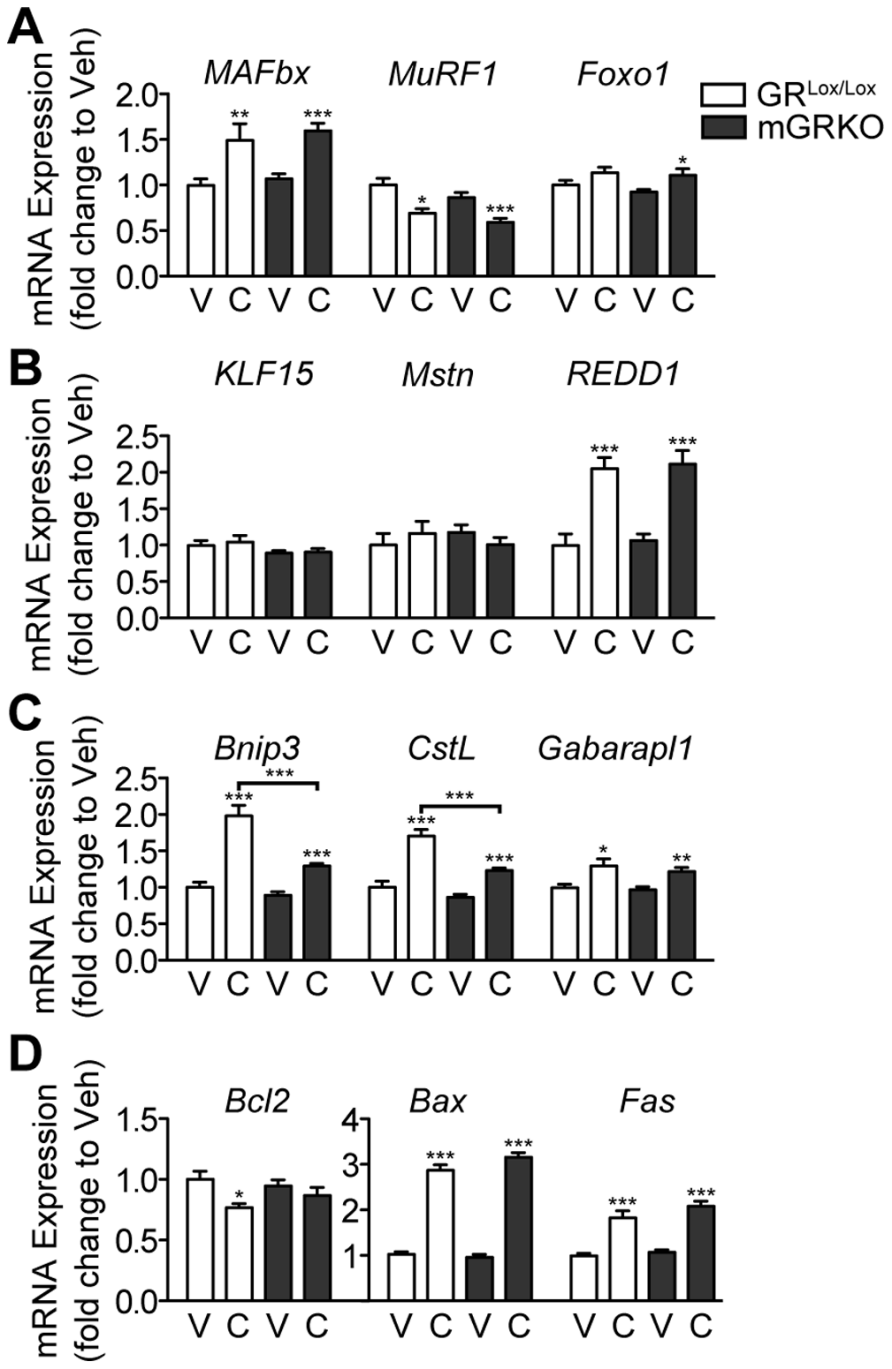
The Gene Expression Signature of Chemotherapy-Induced Cardiotoxicity Contains Glucocorticoid Dependent and Independent Elements. GR^Lox/Lox^ and mGRKO mice (n = 6–13/group) were treated with CAF chemotherapy and sacrificed 18 hours later. (A, B, C, D) Heart gene expression was measured by quantitative real time PCR with GAPDH as an endogenous control. * = P<0.05, ** = P<0.01, and *** = P<0.001 vs. sham-treated mice of the same genotype as measured by two-way ANOVA with Bonferroni post-test. V = Vehicle, C = Chemotherapy.

Wild type C57BL/6J mice and MCK Cre mice were obtained from The Jackson Laboratories (Bar Harbor, ME, USA). GR^Lox/Lox^ mice were provided by L. Muglia (Washington University, St. Lois, MO, USA). All animals were maintained on a normal 12:12h light dark cycle and provided *ad libitum* access to water and food (Purina rodent diet 5001; Purina Mills, St. Louis, MO, USA). Animals were used for experimentation between 6 and 20 weeks of age, and were age, sex, and weight matched in all experiments. No effect of age, sex or weight was observed in any experiments. Mice were first given an intraperitoneal (i.p.) injection with a combination of cyclophosphamide (Baxter Healthcare Corporation, Deerfield, IL, USA), doxorubicin (Bedford Labs, Bedford, OH, USA) at concentrations of 167 mg/kg and 4 mg/kg, respectively. This was then followed by i.p. injection of 5-fluorouracil (5-FU) (American Pharmaceutical Partners, Schaumburg, IL, USA) one hour later at a concentration of 167 mg/kg, as mixing results in rapid drug precipitation [20]. Control mice were administered an equivalent volume of vehicle at these time points. Mice were euthanized by ketamine/xylazine injection. Animals sacrificed at 4 h after the second chemotherapy injection (Figure 1, Table S1) were utilized for circulating cytokine and corticosterone analysis as well as tissue cytokine expression. Animals sacrificed at 18 h were utilized for the assessment of muscle atrophy (Figure 2–5). Blood was obtained by cardiac puncture, anticoagulated with EDTA and separated by centrifugation. Plasma was stored at −80°C for further analysis. Tibialis anterior muscles were removed, frozen in isopentane/LN_2_ and stored at −80°C for histologic analysis. Gastrocnemius muscles were removed and weighed. One gastrocnemius muscle was stored in RNALater (Life Technologies, Carlsbad, CA, USA) according to the manufacturers instructions. The other gastrocnemius was snap frozen in LN_2_ and stored at –80°C for protein analysis.

### Quantitative Real Time PCR

Total muscle RNA was extracted with an RNeasy fibrous tissue mini kit (Qiagen, Valencia, CA, USA) according to the manufacturer’s instructions. cDNA was transcribed with Taqman reverse transcription reagents and random hexamers according to the manufacturers instructions (Life Technologies, Carlsbad, CA, USA). PCR reactions were run on an ABI 7300, with Taqman universal PCR master mix, and Taqman gene expression assays: mouse GAPDH (Mm99999915_g1), mouse *MAFbx* (Mm00499518_m1), mouse *MuRF1* (Mm01185221_m1), mouse *Foxo1* (Mm00490672_m1), Mouse *REDD1* (Mm00512504_g1), Mouse *KLF15* (Mm00517792_m1), Mouse *Mstn* (Mm01254559_m1), Mouse *Bnip3* (Mm01275600_g1), Mouse *CstL* (Mm00515597_m1), Mouse *Gabarapl1* (Mm00457880_m1), mouse TNF (Mm00443260_g1), mouse IL-10 (Mm99999062_m1), mouse IL-18 (Mm00434225_m1), mouse IL-1b (Mm01336189_m1), mouse IL-6 (Mm00446190_m1), mouse Fas (Mm00433237_m1), mouse Bax (Mm00432050_m1), mouse Bcl2 (Mm00477631_m1) (Life Technologies, Carlsbad, CA, USA). Relative expression was calculated via the delta-delta Ct method, and was normalized to vehicle treated control. Statistical analysis was performed on the normally distributed delta-Ct values.

### Immunohistochemistry

Cryosections of the tibials anterior (TA) were cut in cross section at 10µm. Unfixed cryosections were blocked for 1 hour in PBS (10 mM NaPO4, 150 mM NaCl)/1% BSA/10% goat serum, and then incubated overnight in primary antibodies diluted 1:250 in PBS/1% BSA/10% goat serum. The following mouse primary antibodies were used: SC-71 for myosin IIa, BF-F3 for myosin IIb, BA-D5 for myosin I (Developmental Studies Hybridoma Bank, University of Iowa, Iowa City, Iowa, USA). Basement membrane was identified by staining with a rabbit anti-laminin antibody (Sigma, St Louis, MO, USA). Sections were washed in PBS, and incubated with the secondary antibodies diluted 1:500 in 1%BSA/10% goat serum. The following secondary antibodies were used: goat anti rabbit Alexafluor 405, goat anti mouse IgM Alexafluor 488, goat anti mouse IgG2b Alexafluor 555, and goat anti mouse IgG1 Alexafluor 633 (Life Technologies, Carlsbad, CA, USA). Sections were washed in PBS and mounted with aqua-poly mount (Polysciences, Warrington, PA, USA). Images were acquired on a Nikon Eclipse Ti confocal fluorescence microscope (Nikon Instruments Inc., Melville, NY, USA). Tiled images of entire sections were captured at ambient temperature and at 200X. Images were processed in Image J (National Institutes of Health, Bethesda, MD, USA). An evenly spaced grid was placed over the section. Fiber area was measured in fibers expressing Myosin IIb, Myosin IIa, or in those lacking expression of Myosin IIb, IIa and Myosin I (type IId/x fibers) at grid crossing points along with all adjacent fibers of the same type. Grid size was adjusted for a given fiber type such that ∼100 fibers were counted per section. Representative images were selected from the same anatomic region of the TA from animals representative of the group mean fiber area.

### Western Blotting

Muscles were homogenized in cell lysis buffer (Cell Signaling, Danvers, MA): 20 mM Tris pH 7.5, 150 mM NaCl, 1 mM EDTA, 1 mM EGTA 1% Triton X, 2.5 mM sodium pyrophosphate, 1 mM beta-glycerophosphate, 1 mM Sodium Orthovanadate, 1ug/mL leupeptin, and supplemented with Complete protease inhibitors (Roche, Indianapolis, IN), 1 mM PMSF (Sigma, St Louis, MO), and 5 µL/mL phosphatase inibitor cocktail II (Sigma, St Louis, MO). Samples were homogenized for 30 s using a Polytron homogenizer (Kinematica, Bohemia, NY), then were sonicated 2×10 s. Samples were then centrifuged at 13,000 RPM for 10 minutes at 4C. For western blots, 100 ug of total protein per lane was run on 10–20% gradient tris-glycine polyacrylamide gels (Life Technologies, Carlsbad, CA, USA), and transferred to Immobilon PVDF-FL membranes (Millipore, Billerica, MA). Proteins were detected using an Odyssey imaging system (Licor, Lincoln, NE). The following antibodies were used: rabbit anti-LC3b (Cell Signaling, Danvers, MA) and anti-rabbit Dylitght 680 (Cell Signaling, Danvers, MA).

### Corticosterone Measurement

Plasma corticosterone levels were measured by RIA (MP Biomedicals, Solon, OH) according to the manufacturer’s instructions.

### Serum Cytokine Analysis

Serum cytokine levels were assessed using a multiplex magnetic bead based assay according to the manufacturers instructions (Life Technologies, Grand Island, NY). Serum levels of all cytokines on the panel are reported except for, Interleukin-13, Interleukin-1α, and Granulocyte-Macrophage Colony Stimulating Factor, which were below the limits of detection. In many cases samples fell below the lowest portion of the standard curve. When this occurred, but signal was still detectable, a value of the lowest point on the standard curve was assigned. When signal was undetectable, a zero was utilized. This analysis method resulted in data that did not follow a Gaussian distribution. Therefore, in these cases, the non-parametric Mann-Whitney test was utilized for statistical analysis of differential cytokine levels.

### Statistical Analysis

Data are expressed as mean ±SEM. Statistical analysis was performed with Prism software (Version 4.0, Prism Software Corp., Irvine, CA, USA). All data were analyzed with a two-way ANOVA followed with *post hoc* analysis with a Bonferroni post-test or Students t-test as appropriate. For all analyses, significance was assigned at the level of P<0.05.

## Results

### Chemotherapy increases circulating corticosterone and systemic inflammation

To investigate whether chemotherapy induces a systemic inflammatory response, we treated wild type mice with CAF, a common chemotherapy regimen utilized in breast cancer that contains the anthracycline doxorubicin as well as the nucleoside analogue 5-fluorouracil and the alkylating agent cyclophosphamide. The mice were sacrificed 4 hours after injection and examined for evidence of inflammation as well as increases in circulating corticosterone, the predominant glucocorticoid in rodents. Chemotherapy treatment resulted in a significant increase in serum levels of corticosterone (Figure 1 A). In accordance with this, chemotherapy also induced an inflammatory response in the hypothalamus and spleen. IL-6, a known activator of the HPA axis [21], was significantly increased in expression after chemotherapy administration (Figure 1 B). In the spleen, there was a trend toward increased IL-6 expression that did not rise to the level of statistical significance (Figure 1 C). However, there was a significant increase in the expression of IL-10, which is associated with the development of cachexia [22]. We also examined circulating cytokine levels after chemotherapy administration by multiplex bead assay (Table S1). While there was a trend toward increased IL-10 levels after chemotherapy, this did not rise to the level of statistical significance. Interestingly, circulating levels of TNF and IL-5 both decreased significantly 4 h after chemotherapy administration. Collectively, these data demonstrate that the administration of chemotherapy results in significant modulation of systemic inflammatory tone and an increase in serum corticosterone levels.

### Glucocorticoid signaling in skeletal muscle is required for muscle mass loss in response to chemotherapy administration

A large component of the catabolic stimulus in response to systemic inflammation is driven by increases in circulating glucocorticoids [4], [12], [13]. Given that CAF potently induces corticosterone, we investigated whether glucocorticoids contribute to muscle atrophy in response to chemotherapy. To address this question, we utilized muscle-specific glucocorticoid receptor knockout mice (mGRKO), which have recently been shown to resist muscle atrophy in response to tumor growth and acute inflammation [4]. Both mGRKO and GR^Lox/Lox^ littermate control mice were administered CAF chemotherapy and sacrificed 18 h later. Treatment with CAF resulted in significant decrease in food intake and body weight in both genotypes of mice (Figure 2 A, B). However, while muscle mass was significantly reduced in GR^Lox/Lox^ mice in response to chemotherapy, mGRKO mice were completely protected demonstrating that glucocorticoid signaling in muscle is requisite for chemotherapy-induced atrophy (Figure 2 C, D).

### Chemotherapy induces genes involved in the ubiquitin-proteasome and autophagy-lysosome system in skeletal muscle via the action of glucocorticoids

Muscle atrophy occurs, at a molecular level, as the result of the proteasomal degradation of myofibrillar protein. Two muscle-specific E3 ubiquitin ligases, Muscle Atrophy F-box (MAFbx) and Muscle Ring Finger Protein 1 (MuRF1) are rapidly induced in catabolic skeletal muscle and facilitate the ubiquitination of contractile proteins [14], [23]. The transcriptional regulation of these E3 ligases is complex, and includes direct regulation by the glucocorticoid receptor. Additionally, the forkhead box (Foxo) family of transcription factors, Krüpple like factor 15 (KLF15) and myostatin/SMAD signaling also play a critical role [24], [25]. In response to chemotherapy administration in GR^Lox/Lox^ mice, MAFbx, MuRF1 and Foxo1 expression in skeletal muscle are all increased 18 hours after chemotherapy administration (Figure 3 A). However, this response is blunted or entirely absent in mGRKO mice receiving CAF. Interestingly, KLF15 was not significantly induced by chemotherapy, despite a marked induction by exogenous glucocorticoids in other studies [4]. Regulated in Development and DNA Damage Responses 1 (REDD1), a regulator of protein synthesis was significantly induced by chemotherapy in both genotypes of mice (Figure 3 B). Despite playing a critical role in muscle atrophy in response to exogenous glucocorticoid administration, myostatin was not induced by chemotherapy administration [26].

It has recently become appreciated that the autophagy-lysosome system plays a fundamental role in the process of muscle atrophy [15], [27]. We therefore investigated in expression of the autophagy genes BCL-2/adenovirus E1B-19kDA-interacting protein 3 (Bnip3), and GABA(A) receptor-associated protein like 1(Gabarapl1) along with the lysosomal protease Cathepsin L (CstL), which are regulated transcriptionally and consistently elevated in muscle undergoing atrophy [4], [12], [15]. Chemotherapy significantly induced Bnip3 and Gabapl1 GR^Lox/Lox^ mice. However, this response was partially blocked in mGRKO mice, where the induction of Bnip3 by chemotherapy was significantly attenuated (Figure 3 C). We also assessed the conversion of light chain 3 B (LC3B)-I to LC3B-II, a process involving lipidation that is required for formation of autophagic vesicles. Consistent with prior work, mice treated with LPS demonstrate a significant increase in the 14 kD LC3B-II isoform (Figure S1) [28]. However, chemotherapy failed to induce a significant conversion of LC3B-I to LC3B-II in either genotype of mice, consistent with the relatively weak induction of autophagy genes relative to LPS treatment [4].

Skeletal muscle is also a known producer of inflammatory cytokines. Therefore, we investigated whether chemotherapy administration increases muscle cytokine production and whether this effect is dependent on glucocorticoid signaling. In response to chemotherapy administration, there was a significant induction of IL-1β in the muscle of mGRKO but not in GR^Lox/Lox^ mice consistent with the role of glucocorticoids as a negative regulator of inflammation (Figure 3 D). Chemotherapy failed to induce IL-6 or TNF expression in the muscle of either genotype of mouse.

Collectively, these data demonstrate that glucocorticoids are required for the induction of the catabolic program in response to chemotherapy administration. The most pronounced changes occur in genes regulating proteasomal degradation of skeletal muscle, while the effects on other pathways such as the autophagy lysosome system are relatively modest compared with other catabolic stimuli. While implicated in other forms of muscle atrophy, the transcriptional regulation of myostatin or the production of inflammatory cytokines by muscle does not occur in response to chemotherapy administration.

### Muscle atrophy in response to chemotherapy is dependent on glucocorticoid signaling in skeletal muscle

To investigate the role of glucocorticoids of producing myofibrillar atrophy, tibialis anterior muscle from GR^Lox/Lox^ and mGRKO mice treated with chemotherapy was cut in cross section and immunostained to delineate fiber type. A marked loss of cross sectional area was seen in the glycolytic type IIb fibers of GR^Lox/Lox^ mice 18 hours after chemotherapy administration (Figure 4 A, B). However, in mGRKO mice, no loss of type IIb fiber cross sectional area was seen. While there was also a trend toward a decrease in the cross sectional area of the more oxidative type IIa and IId/x fibers in response to chemotherapy, this did not rise to the level of statistical significance. This demonstrates that the loss of skeletal muscle mass in response to chemotherapy largely arises from the atrophy of fast twitch glycolytic type IIb fibers.

### Glucocorticoids contribute to induction of pro-apoptotic pathways in cardiac muscle

The MCK-Cre promoter used to generate the muscle-specific glucocorticoid receptor knockout mouse is also active in the heart, and therefore can also be utilized to assess the contribution of glucocorticoid signaling to chemotherapy-induced cardiotoxicity. We therefore examined the expression of atrophy-related genes in cardiac muscle 18 hours after chemotherapy administration (Figure 5 A). MAFbx expression was significantly increased in the hearts of animals receiving chemotherapy irrespective of genotype, while MuRF1 expression was decreased. There was a trend toward increased expression of Foxo1 in cardiac muscle from chemotherapy treated animals, however this only rose to the level of statistical significance in mGRKO mice. The expression of the mTOR regulators KLF15 and REDD1 was also examined (Figure 5 B). While the expression of KLF15 did not change in response to chemotherapy, REDD1 was significantly induced in the heart of both mGRKO mice and GR^Lox/Lox^ littermates. Interestingly, there were some genotype-dependent effects on the chemotherapy-mediated induction of genes controlling autophagy in cardiac muscle (Figure 5 C).

Deletion of glucocorticoid receptor in cardiomyocytes suppressed the increase in Bnip3 and CstL brought about by chemotherapy administration.

Finally, given the extensive literature on chemotherapy-induced cardiomyocyte apoptosis, we investigated the expression of apoptotic regulators in the hearts of mGRKO mice treated with chemotherapy (Figure 5D). Cardiac expression of both Bax and Fas was increased equally by chemotherapy in mGRKO and GR^Lox/Lox^ mice. The anti-apoptotic regulator Bcl2 decreased in expression as a result of chemotherapy, however, this response was significantly attenuated in mGRKO mice, where the reduction occurred to a lesser extent. Collectively these data demonstrate that, unlike skeletal muscle, the majority of the catabolic insult inflicted on cardiac muscle by chemotherapy is not conveyed by glucocorticoids. However, there is a small but significant glucocorticoid-dependent component of this process involving the regulation of genes involved in autophagy and apoptosis.

## Discussion

Cytotoxic chemotherapy induces cellular damage through numerous mechanisms including the generation of oxidative stress and the induction of DNA damage. Given the well established direct effects of chemotherapy on the heart it would be reasonable to postulate an analogous direct cytotoxic effect on skeletal muscle [16]. It was therefore interesting to discover that mGRKO mice are resistant to chemotherapy-induced muscle atrophy, arguing for glucocorticoids as a critical intermediary. This demonstrates that the direct cytotoxic effects of chemotherapy on skeletal muscle are dispensable or at least not sufficient in isolation to drive the process of muscle atrophy.

The finding of localized inflammation after chemotherapy administration is consistent with an extensive literature documenting the activation of inflammatory signaling by chemotherapy. Doxorubicin exposure results in significant IL-1β production via activation of the inflammasome [17], [29]. Others have reported significant increases in IL-6 after chemotherapy administration in cancer patients [30]. In addition to the direct activation of inflammatory signaling pathways, cell death and the release of intracellular damage-associated molecular patterns also likely contribute to the inflammatory response seen after the administration of chemotherapy. Potent immune activators such high mobility group box 1 (HMGB1) are released and signal via toll-like receptors to activate NFκB leading to cytokine production [31]. Interestingly, despite evidence of tissue inflammation and corticosterone release, we did not observe a significant increase in circulating inflammatory cytokines 4 hours after chemotherapy administration. It is possible that circulating cytokines may be elevated at a shorter time point after chemotherapy administration, returning to baseline after 4 hours. Alternately, chemotherapy may produce localized inflammation within the CNS leading to HPA axis activation. Although the direct action of cytokines on skeletal muscle is a well-documented mechanism of atrophy, the data presented here are consistent with a growing body of evidence demonstrating that CNS inflammation is all that is required for HPA axis activation and muscle atrophy [4], [13].

A large body of evidence supports the necessity of NFκB activation in skeletal myocytes for the development of atrophy. NFκB activity is increased in response to multiple atrophic stimuli and genetic blockade of this pathway protects against atrophy in response to denervation as well as tumor growth [6]. Chemotherapy also increases NFκB DNA binding in skeletal muscle and has been proposed the driver of muscle atrophy in this setting [7]. This is seemingly at odds with our finding that glucocorticoid signaling, known to antagonize inflammatory pathways, is required for muscle atrophy in this proinflammatory state [32]. However, our results are consistent with an emerging body of literature demonstrating that under certain physiologic conditions, glucocorticoids potentiate rather than inhibit immune responses. Indeed, it appears that glucocorticoid levels within the physiologic range are permissive for a normal early immune response and only become suppressive at higher doses over longer time courses [33]. Additionally, many of the fundamental studies investigating the anti-inflammatory properties of glucocorticoids have been done using supraphysiologic doses of synthetic ligands with higher receptor affinity, increased half-life and altered mineralocorticoid activity when compared with endogenous glucocorticoids [34]. While a definitive understanding of the relationship between NFκB and glucocorticoid signaling in skeletal muscle awaits a detailed investigation, it is plausible that inflammatory and glucocorticoid signaling cooperatively induce the catabolic process.

The precise role of the Foxo1-MAFbx/MuRF1 axis in cardiac muscle is less clearly defined than it is in skeletal muscle. In contrast to its clearly defined role as a driver of atrophy in skeletal muscle, both MAFbx and MuRF1 are drivers of overload-induced cardiac hypertrophy [35], [36]. However, others have described a catabolic role for MAFbx, where overexpression in myocardium prevents Akt-induced hypertrophy [37]. Interestingly, cardiac MAFbx expression increased after chemotherapy administration, while the expression of MuRF1 declined.

Deletion of the glucocorticoid receptor from cardiac muscle failed to alter the regulation of these genes by chemotherapy. This argues that the regulation of these genes in the myocardium by chemotherapy occurs as the result of direct cytotoxicity or the regulation of an as-of-yet unknown systemic factor.

The induction of autophagy by chemotherapy has been implicated as a mediator of cardiotoxicity [38]. Our results demonstrate that this process is partially dependent on cardiac glucocorticoid signaling, as the deletion of glucocorticoid receptors in the heart attenuates the induction of genes involved in autophagy. Likewise, prior work has demonstrated that the pro-apoptotic effects of anthracycline chemotherapy on cardiomyocytes are the result of the direct interaction with cardiac topoisomerase IIb. While our results demonstrate normal induction of Bax and Fas in the absence of glucocorticoid signaling, the suppression of the anti-apoptotic gene Bcl2 by chemotherapy is attenuated when glucocorticoid signaling is blocked genetically. Given the magnitude of the suppression and the unaltered induction of pro apoptotic factors, it is unclear whether this effect alone carries physiologic significance. However, in combination with the attenuation of the induction of autophagy gene expression, it is likely that glucocorticoids contribute to the cardiotoxicity of chemotherapy.

Our findings are the first to demonstrate a role for glucocorticoids in the development of muscle atrophy after acute administration of cytotoxic chemotherapy. This finding introduces the possibility of therapeutic intervention that can be easily timed to coincide with the administration of chemotherapy. Future studies will likely evaluate the combined effects of chemotherapy and tumor growth on the development of cachexia.

## Supporting Information

Figure S1 **LC3B I:II Interconversion in Skeletal Muscle After CAF Chemotherapy.** GR^Lox/Lox^ and mGRKO mice were treated with CAF chemotherapy and sacrificed 18 hours later. Wild type mice were injected with LPS (1 mg/kg) and sacrificed 18 hours later. LC3B was detected by western blotting in muscle homogenates. Veh = Vehichle, Chemo = CAF chemotherapy.
(PDF)

Table S1 **Circulating Cytokines After CAF Chemotherapy.** Wild type mice (10/group) were treated with CAF chemotherapy and sacrificed 4 hours later. Serum cytokines were assessed by multiplex magnetic bead cytokine assay. Values presented as the mean± SEM. P values obtained via Students t-test or Mann Whitney (indicated by #) test for non-Gaussian distributions as described in the methods. Cytokines listed in bold where P<0.05.
(PDF)
