# Supplementary figures and images for: Muscle Atrophy in Response to Cytotoxic Chemotherapy Is Dependent on Intact Glucocorticoid Signaling in Skeletal Muscle
Source: PLoS One. 2014 Sep 25;9(9):e106489. doi: 10.1371/journal.pone.0106489 (PMC4177815; doi:10.1371/journal.pone.0106489)

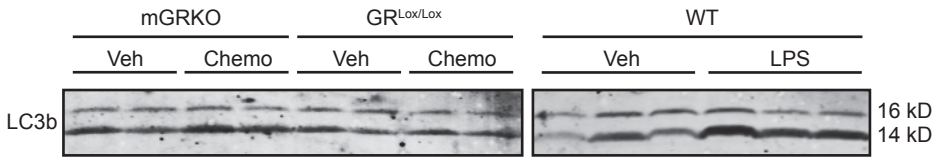

Figure S1

Supplement: Figure S1 — LC3B I:II Interconversion in Skeletal Muscle After CAF Chemotherapy. GRLox/Lox and mGRKO mice were treated with CAF chemotherapy and sacrificed 18 hours later. Wild type mice were injected with LPS (1 mg/kg) and sacrificed 18 hours later. LC3B was detected by western blotting in muscle homogenates. Veh = Vehichle, Chemo = CAF chemotherapy. (PDF) [file pone.0106489.s001.pdf]
