# Supplementary material for: Muscle Atrophy in Response to Cytotoxic Chemotherapy Is Dependent on Intact Glucocorticoid Signaling in Skeletal Muscle
Source: PLoS One. 2014 Sep 25;9(9):e106489. doi: 10.1371/journal.pone.0106489 (PMC4177815; doi:10.1371/journal.pone.0106489)
Supplement: Table S1 — Circulating Cytokines After CAF Chemotherapy. Wild type mice (10/group) were treated with CAF chemotherapy and sacrificed 4 hours later. Serum cytokines were assessed by multiplex magnetic bead cytokine assay. Values presented as the mean± SEM. P values obtained via Students t-test or Mann Whitney (indicated by #) test for non-Gaussian distributions as described in the methods. Cytokines listed in bold where P<0.05. (PDF) [file pone.0106489.s002.pdf]

Cytokine	Sham (pg/mL)	Chemotherapy (pg/mL)	P value
IL-1β	5.9± 1.3	5.5± 1.24	0.88#
IL-2	5.9± 1.4	5.4± 1.4	0.59#
IL-4	13.6± 5.0	8.9± 2.1	0.88#
**IL-5**	**36.0± 4.9**	**18.3± 4.5**	**0.017**
IL-6	24.8± 5.3	31.4± 15.9	0.70
IL-10	26.5± 7.9	66.8± 30.2	0.30#
IL-12	49.6± 8.9	38.9± 6.4	0.34
IL-17	2.0± 0.99	0.83± 0.16	0.82#
FGF basic	102.5± 11.9	98.0± 9.2	0.44#
CXCL10	40.4± 11.8	63.9± 20.8	0.34
IFN γ	11.9±2.8	9.2± 2.6	0.45#
MIG	43.7± 25.4	39.5± 29.6	0.91
**TNF**	**10.1±1.5**	**5.1± 1.7**	**0.0068#**
VEGF	3.4± 0.2	3.4± 0.3	0.98
CCL2	21.9± 5.7	10.5± 3.7	0.063#
MIP1a	135.4± 43.4	65.4± 17.4	0.15
KC	260.0± 111.5	361.5± 124.2	0.94#

Table S1
